# Synaptosomal Protein of 25 kDa (*Snap25*) Polymorphisms Associated with Glycemic Parameters in Type 2 Diabetes Patients

**DOI:** 10.1155/2016/8943092

**Published:** 2015-12-08

**Authors:** Nasser M. Al-Daghri, Andrea S. Costa, Majed S. Alokail, Milena Zanzottera, Amal M. Alenad, Abdul Khader Mohammed, Mario Clerici, Franca R. Guerini

**Affiliations:** ^1^Biomarkers Research Program, Biochemistry Department, College of Science, King Saud University, Riyadh 11451, Saudi Arabia; ^2^Prince Mutaib Chair for Biomarkers of Osteoporosis, Biochemistry Department, College of Science, King Saud University, Riyadh 11451, Saudi Arabia; ^3^Fondazione Don C Gnocchi, IRCCS, 20148 Milano, Italy; ^4^School of Biological Sciences, University of Southampton, Southampton SO17 1 BJ, UK; ^5^Università degli Studi di Milano, 20122 Milano, Italy

## Abstract

A possible role of* Snap25* polymorphisms in type 2 diabetes mellitus (T2DM) was evaluated by analyzing three SNPs within intron 1 in a region known to affect the gene expression in vitro. Genomic DNA from 1019 Saudi individuals (489 confirmed T2DM and 530 controls) was genotyped for SNPs rs363039, rs363043, and rs363050 in* Snap25* using the TaqMan Genotyping Assay. Significantly higher levels of fasting glucose and HbA1c were detected in T2DM patients carrying the rs363050 (AG/GG) genotypes compared to the (AA) genotype (*f* = 4.41, *df* = 1, and *p* = 0.03 and *f* = 5.31, *df* = 1, and *p* = 0.03, resp.). In these same patients, insulin levels were significantly decreased compared to the (AA) individuals (*f* = 7.29, *df* = 1, and *p* = 0.009). Significant associations were detected between rs363050 (AG/GG) genotypes and increasing fasting glucose levels (*p* = 0.01 and OR: 1.05), HbA1c levels (OR: 5.06 and *p* = 0.02), and lower insulinemia (*p* = 0.03 and OR: 0.95) in T2DM patients. The minor* Snap25* rs363050 (G) allele, which results in a reduced expression of* Snap25*, is associated with altered glycemic parameters in T2DM possibly because of reduced functionality in the exocytotic machinery leading to suboptimal release of insulin.

## 1. Introduction

Blood glucose levels are regulated by insulin release from pancreatic *β*-cells via exocytosis, a fine-tuned mechanism mediated by complex machinery. During the exocytotic process secretory granules (SG) of insulin fuse with the plasma membrane; this process is mediated by a number of key players including the soluble* N*-ethylmaleimide-sensitive factor attachment protein receptor (SNARE) proteins. The SNARE-complex includes the two t-SNARE proteins, synaptosomal protein of 25 kDa (Snap25) and syntaxin 1A, as well as the v-SNARE protein VAMP2. To allow exocytosis the amino terminal of Snap25 binds to syntaxin 1A and the carboxy-terminal binds to VAMP2, forming the four-helical bundle that brings SG in close contact with the plasma membrane, thus enabling fusion to occur. Snap25 is contained in insulin-secreting cells [1] and is known to modulate several processes apart from the actual fusion event, including the activity of potassium voltage gated (Kv)2.1 channels [2]. Notably, Kv2.1 is the prevalent Kv channel in neuroendocrine and endocrine cells, and the Kv2.1 current repolarizes *β*-cell action potentials upon exposure to glucose to limit Ca^2+^ entry and insulin secretion.

Because of the pivotal role of Snap25 in insulin release [2–6] we evaluated a possible role of three* Snap25* polymorphisms (rs363039, rs363043, and rs363050) that are localized within intron 1 in a region spanning about 13.8 kb and are known to affect the gene expression in vitro [7, 8], in type 2 diabetes mellitus (T2DM). To this end, fasting glycemia, insulinemia, and the concentration of glycated haemoglobin (HbA1c) were correlated with* Snap25* polymorphisms in 489 patients with a diagnosis of type 2 diabetes mellitus (T2DM) and 530 age- and sex-matched healthy controls (HC).

## 2. Methods

### 2.1. Patients and Controls

A total of 1019 unrelated adult Saudi individuals (489 confirmed T2DM (280 males and 209 females) and 530 controls (319 males and 211 females)) were randomly selected from the Biomarker Screening Project in Riyadh (Riyadh Cohort), a capital-wide epidemiological study of over 17,000 consenting Saudis coming from different Primary Health Care Centers (PHCCs) in Kingdom of Saudi Arabia (KSA). Written informed consent was obtained from all participants before inclusion in the study. The research was approved by the Ethics Committee of the College of Science, King Saud University, Riyadh, KSA, and the methods were carried out in accordance with the approved guidelines. Diagnosis of T2DM was based on World Health Organization (WHO) proposed cut-off: fasting plasma glucose > or = 7.0 mmol/L or 126 mg/dL. Subjects with comorbidities that needed medical attention or with medical complications (coronary artery disease, nephropathy, thyroid diseases, and end stage renal or liver disease) were excluded from the study. Consenting participants were then requested to visit the nearest participating PHCC for questionnaire administration, anthropometric measurement, and blood extraction. A questionnaire focusing on demographic information and past and present medical history was given to all subjects.

### 2.2. Anthropometry, Blood Collection, and Biochemical Analyses

Subjects were requested to visit their respective PHCCs following overnight fasting (>10 hours) for anthropometry and blood withdrawal. Anthropometry included height (rounded off to the nearest 0.5 cm), weight (rounded off to the nearest 0.1 kg), waist and hip circumference (centimeters), and mean systolic and diastolic blood pressure (millimeters of Hg) (average of 2 readings). Body mass index (BMI) was calculated as weight in kilograms divided by height in square meters. Fasting glucose and lipid profile were measured using a chemical analyzer (Konelab, Espoo, Finland). Serum insulin was assessed using an enzyme-linked immunosorbent assay (ELISA) with an intra-assay variability of <10% and interassay variation of <15%. All fasting samples fell within the detection range.

### 2.3. *Snap25 *Genotyping

Genomic DNA was isolated from whole blood using the blood genomicPrep mini spin kit (GE Healthcare Life Sciences, Piscataway, NJ, USA). SNPs rs363043, rs363039, and rs363050 located in intron 1 of* Snap25* gene were genotyped using the TaqMan SNP Genotyping Assays (Applied Biosystems by Life Technologies, Foster City, CA, USA) on an ABI PRISM 7000 Sequence Detection System.

### 2.4. Statistical Analysis

The Kolmogorov-Smirnov (K-S) test was applied to verify normal distribution of numerical variable scores. All numerical scores resulted in being normally distributed, and they were shown as mean and standard deviation (SD). Hardy-Weinberg equilibrium was tested using Chi-square test. ANOVA calculation was performed to analyse biochemical parameters in relationship with* Snap25* genotypes stratified for T2DM and HC. Moreover multivariate stepwise logistic regression analysis was performed, using* Snap25* polymorphisms as responsible variables and age, gender, and BMI as covariates.

## 3. Results and Discussion

Fasting glycemia, insulinemia, and the concentration of glycated haemoglobin (HbA1c) were correlated with* Snap25* polymorphisms in 489 patients with a diagnosis of T2DM and 530 age- and sex-matched HC (Table 1), whereas no differences were seen in the distribution of the 3* Snap25* SNPs between T2DM and controls (data not shown). Significantly higher levels of fasting glucose and HbA1c were detected in T2DM patients carrying the rs363050 (AG/GG) (57% of cases) genotypes compared to those carrying the (AA) genotype (43% of cases) (*f* = 4.41, *df* = 1, and *p* = 0.03 and *f* = 5.31, *df* = 1, and *p* = 0.03, resp.). In these same patients insulin levels were significantly decreased as well compared to the (AA) individuals (*f* = 7.29,  *df* = 1, and *p* = 0.009) (Table 2). In contrast with these results, no associations were observed between any of the* Snap25* genotypes and glycemic parameters in HC (*f* = 0.88,  *df* = 1, and *p* = 0.35).

The correlation between the rs363050 (G) minor allele and altered glycemic parameters in T2DM patients was even stronger when multivariate stepwise logistic regression analysis was performed. Thus, when rs363050 (AG/GG) versus (AA) was calculated as the responsible variable and age, gender, and BMI were calculated as covariates, a statistically significant association was detected between (AG/GG) genotypes and increasing fasting glucose levels (*p* = 0.01, OR: 1.05), with a higher risk in female versus male patients (*p* = 0.03, OR: 1.63). A similar association was observed as well between the (AG/GG) genotypes and increasing HbA1c levels (*p* = 0.02, OR: 5.06) and between these genotypes and lower insulinemia (*p* = 0.03, OR: 0.95) in T2DM patients after adjustment for BMI, gender, and age covariates, even if in this case no specific risk was detected in female patients. Again, no such associations were observed in healthy controls. To rule out a possible role played by confounding factors, diabetes duration, hypoglycemic drug use, insulin resistance condition, and anthropometric parameters were compared between patients carrying the two genotypes, and no significant differences emerged (data not shown).

Insulin secretion depends on the integrity of the SNARE exocytotic machinery, as shown by the observation that decreased expression of t-SNARE associates with an alteration of this process [5].  Snap25, a protein that is expressed in pancreatic endocrine cells, is an essential component of such machinery [4]. Data herein shows that the* Snap25* rs363050 (G) allele correlates with increasing glycemia and HbA1c concentration, as well as with reduced insulinemia in T2DM patients alone; this allele was recently demonstrated to be associated with a significantly reduced gene expression in vitro compared to the rs363050 (A) allele [8]. Because no associations between glycemic parameters and* Snap25* polymorphism were detected in healthy controls this polymorphism is not likely to be a genetic risk factor for T2DM by itself but rather to correlate with altered T2DM-related metabolic parameters once T2DM has been diagnosed. This effect is possibly mediated by impairment in the SNARE complex, resulting in a reduced functionality of the exocytotic machinery and, as a consequence, in a suboptimal release of insulin.

## 4. Conclusions

Snap25 plays an important role in exocytosis, and its polymorphisms have been implicated in modulating the release of neurotransmitters in a number of neurologic conditions including autism and Alzheimer's disease (AD) [9]. Importantly, the integrity of the insulin signaling plays a pivotal role for cognitive function and memory. Thus, in neurons, insulin stimulates growth, survival, differentiation, metabolism, protein synthesis, synapse formation, and plasticity [10]. The preliminary results herein indicate that the same* Snap25* SNPs are present in AD and in T2DM; these data add further evidence to the importance played by an intact insulin signaling pathway in the maintenance of well-structured cognitive functions and reinforce the idea that* Snap25* polymorphisms play a role in metabolic disorders. Notably, polymorphisms in the human* Snap25* gene have been associated with weight gain after antipsychotic treatment and with altered levels of serum triglycerides [11, 12] and, importantly, with the severity of metabolic syndrome in T2DM [13] These data, finally, confirm previous results showing a role of Snap25 in insulin secretion [2–6]. Because the incidence of AD is increased in T2DM [14] it will be interesting to explore whether* Snap25* alleles-mediated exocytosis alterations bridge these two pathologies, possibly predicting those T2DM patients with a higher risk of developing AD.

## Figures and Tables

**Table 1:** Clinical characteristic of the T2DM patients and the healthy controls enrolled in the study.

	Healthy controls	T2DM
*N*	530	489
Gender male/female	319/211	280/209
Age (years)	48.5 ± 12.5	51.2 ± 11.5
Systolic BP (mmHg)	117.6 ± 12.3	129.7 ± 15.1^*∗*^
Diastolic BP (mmHg)	75.7 ± 8.4	80.7 ± 8.9^*∗*^
BMI (kg/m^2^)	28.8 ± 7.4	32.0 ± 5.9^*∗*^
Hip circumference (cm)	87.1 ± 22.0	98.0 ± 24.2^*∗*^
Waist circumference (cm)	98.5 ± 25.0	103.0 ± 26.9^*∗*^
Total cholesterol (mmol/L)	5.1 ± 1.0	5.3 ± 1.2^*∗*^
Fasting glucose (mmol/L)	5.1 ± 0.9	12.0 ± 4.7
HbA1c (IU)	5.2 ± 0.5	7.5 ± 1.3^*∗*^
Insulinemia *µ*U/mL	11.2 ± 18.2	18.4 ± 17.7^*∗∗*^
LDL-cholesterol (mmol/L)	3.4 ± 0.9	3.5 ± 1.0
HDL-cholesterol (mmol/L)	0.9 ± 0.4	0.8 ± 0.3^*∗*^
Triglycerides (mmol/L)	1.5 ± 0.8	2.2 ± 1.3^*∗*^

Median values ± standard deviation (SD) are shown. Statistically significant differences are indicated: ^*∗*^ *p* < 0.001; ^*∗∗*^ *p* < 0.05.

**Table 2:** Glycemic parameters in 489 T2DM patients according to the *Snap25* rs36305 genotype.

	*Snap25* rs363050 genotype	
	AA	AG + GG
Fasting glucose (mmol/L)	11.5 ± 4.3^*∗∗*^	12.9 ± 4.9^*∗∗*^
HbA1c (IU)	6.9 ± 0.9^*∗∗*^	8.2 ± 1.4^*∗∗*^
Insulinemia *µ*U/mL	23.9 ± 23.1^*∗*^	13.4 ± 8.1^*∗*^

Median values ± standard deviation (SD) are shown. Statistically significant differences are indicated: ^*∗*^ *p* < 0.001; ^*∗∗*^ *p* < 0.05.

## References

[1] Vikman J., Svensson H., Huang Y.-C. (2009). Truncation of SNAP-25 reduces the stimulatory action of cAMP on rapid exocytosis in insulin-secreting cells. *The American Journal of Physiology—Endocrinology and Metabolism*.

[2] MacDonald P. E., Wang G., Tsuk S. (2002). Synaptosome-associated protein of 25 kilodaltons modulates Kv2.1 Voltage-dependent K^+^ channels in neuroendocrine islet *β*-cells through an interaction with the channel N terminus. *Molecular Endocrinology*.

[3] Sadoul K., Lang J., Montecucco C. (1995). SNAP-25 is expressed in islets of Langerhans and is involved in insulin release. *Journal of Cell Biology*.

[4] Gonelle-Gispert C., Halban P. A., Niemann H., Palmer M., Catsicas S., Sadoul K. (1999). SNAP-25a and -25b isoforms are both expressed in insulin-secreting cells and can function in insulin secretion. *Biochemical Journal*.

[5] Nagamatsu S., Nakamichi Y., Yamamura C. (1999). Decreased expression of t-SNARE, syntaxin 1, and SNAP-25 in pancreatic *β*-cells is involved in impaired insulin secretion from diabetic GK rat islets: restoration of decreased t-SNARE proteins improves impaired insulin secretion. *Diabetes*.

[6] Zhuang G.-Q., Wu W., Liu F. (2009). SNAP-25_1-180_ enhances insulin secretion by blocking Kv2.1 channels in rat pancreatic islet *β*-cells. *Biochemical and Biophysical Research Communications*.

[7] Gosso M. F., De Geus E. J. C., Polderman T. J. C., Boomsma D. I., Heutink P., Posthuma D. (2008). Common variants underlying cognitive ability: further evidence for association between the SNAP-25 gene and cognition using a family-based study in two independent Dutch cohorts. *Genes, Brain and Behavior*.

[8] Braida D., Guerini F. R., Ponzoni L. (2015). Association between SNAP-25 gene polymorphisms and cognition in autism: functional consequences and potential therapeutic strategies. *Translational Psychiatry*.

[9] Guerini F. R., Agliardi C., Sironi M. (2014). Possible association between SNAP-25 single nucleotide polymorphisms and alterations of categorical fluency and functional MRI parameters in Alzheimer's disease. *Journal of Alzheimer's Disease*.

[10] de la Monte S. M. (2012). Brain insulin resistance and deficiency as therapeutic targets in Alzheimer's disease. *Current Alzheimer Research*.

[11] Müller D. J., Klempan T. A., De Luca V. (2005). The SNAP-25 gene may be associated with clinical response and weight gain in antipsychotic treatment of schizophrenia. *Neuroscience Letters*.

[12] Musil R., Spellmann I., Riedel M. (2008). SNAP-25 gene polymorphisms and weight gain in schizophrenic patients. *Journal of Psychiatric Research*.

[13] Chen Y. L., Pei D., Hung Y. J. (2015). Associations between genetic variants and the severity of metabolic syndrome in subjects with type 2 diabetes. *Genetics and Molecular Research*.

[14] Kopf D., Frölich L. (2009). Risk of incident Alzheimer's disease in diabetic patients: a systematic review of prospective trials. *Journal of Alzheimer's Disease*.

